# Sex differences in primary muscle afferent sensitization following ischemia and reperfusion injury

**DOI:** 10.1186/s13293-017-0163-5

**Published:** 2018-01-03

**Authors:** Jessica L. Ross, Luis F. Queme, Jordan E. Lamb, Kathryn J. Green, Michael P. Jankowski

**Affiliations:** 1 Department of Anesthesia, Division of Pain Management, Cincinnati Children’s Hospital Medical Center, 3333 Burnet Ave MLC 6016, Cincinnati, OH 45229 USA; 2 Department of Pediatrics, University of Cincinnati, Cincinnati, OH 45229 USA

**Keywords:** Ischemia, Nociception, Muscle afferents, Behavior, Molecular biology

## Abstract

**Background:**

Chronic pain conditions are more prevalent in women, but most preclinical studies into mechanisms of pain generation are performed using male animals. Furthermore, whereas group III and IV nociceptive muscle afferents provoke central sensitization more effectively than their cutaneous counterparts, less is known about this critical population of muscle nociceptors. Here, we compare the physiology of individual muscle afferents in uninjured males and females. We then characterize the molecular, physiological, and behavioral effects of transient ischemia and reperfusion injury (I/R), a model we have extensively studied in males and in females.

**Methods:**

Response properties and phenotypes to mechanical, thermal, and chemical stimulation were compared using an ex vivo muscle/nerve/dorsal root ganglia (DRG)/spinal cord recording preparation. Analyses of injury-related changes were also performed by assaying evoked and spontaneous pain-related behaviors, as well as mRNA expression of the affected muscle and DRGs. The appropriate analyses of variance and post hoc tests (with false discovery rate corrections when needed) were performed for each measure.

**Results:**

Females have more mechanically sensitive muscle afferents and show greater mechanical and thermal responsiveness than what is found in males. With I/R, both sexes show fewer cells responsive to an innocuous metabolite solution (ATP, lactic acid, and protons), and lower mechanical thresholds in individual afferents; however, females also possess altered thermal responsiveness, which may be related to sex-dependent changes in gene expression within the affected DRGs. Regardless, both sexes show similar increases in I/R-induced pain-like behaviors.

**Conclusions:**

Here, we illustrate a unique phenomenon wherein discrete, sex-dependent mechanisms of primary muscle afferent sensitization after ischemic injury to the periphery may underlie similar behavioral changes between the sexes. Furthermore, although the group III and IV muscle afferents are fully developed functionally, the differential mechanisms of sensitization manifest prior to sexual maturity. Hence, this study illustrates the pressing need for further exploration of sex differences in afferent function throughout the lifespan for use in developing appropriately targeted pain therapies.

**Electronic supplementary material:**

The online version of this article (10.1186/s13293-017-0163-5) contains supplementary material, which is available to authorized users.

## Background

Pain is a significant problem in the USA [1]. The diffuse and subjective properties [2, 3], as well as the heterogeneous etiologies [4], of muscle pain complicate effective management. One particularly nefarious cause of myalgia arises from deficits in peripheral perfusion, where transient ischemia prevents adequate blood flow and oxygen from reaching the muscles [5–7]. This occurs in conditions such as complex regional pain syndrome (CRPS) [8–10], peripheral vascular disease [11, 12], sickle cell anemia [13], and fibromyalgia [14–16]. Clinical features of ischemic myalgia include decreased activity, ongoing pain, hypersensitivity, and weakness in the affected muscle tissue [17]. Correspondingly, animal models of ischemic myalgia display similar enhancements in muscle pain-like behaviors, which correlate with distinct changes in neuronal gene expression and function [9, 18–26] at multiple levels within the canonical pain pathway.

Until recently, these studies have been performed primarily in male rodents, which is translationally counterintuitive as many chronic musculoskeletal pain conditions, including CRPS and fibromyalgia [5, 27], are more prevalent in women [28–30]. Furthermore, there are sex-dependent effects on disease severity and patient outcomes in ischemic myalgia-associated conditions [31–34]. Because of the lack of effective therapies for ischemic myalgia, understanding how deficits in peripheral perfusion generate this type of muscle pain is crucial. To investigate these underlying mechanisms, we have established a mouse model of transient ischemia and reperfusion (I/R) injury to the forepaw muscles, but until now, have only characterized its effects in male mice [19, 35].

Recent studies examining various rodent pain models have suggested that discrete immune mechanisms contribute to sex-dependent sensitization within the spinal cord dorsal horn and brain [36–39], but examinations into sex effects on primary muscle afferents have been limited. The group III and IV afferents that innervate the muscle tissue are responsive to mechanical, thermal, and chemical stimulation, and it has been previously shown that solutions that contain all three of the common muscle “metabolites” (lactic acid, ATP, and protons) are more effective in provoking afferent chemical responses than any one or two alone [40, 41].

Using natural mechanical, thermal, and metabolite stimuli in our novel ex vivo electrophysiology preparation allows us to phenotype individual group III and IV primary muscle afferents, and thus, analyze injury-induced changes in distinct subpopulations [19, 23, 35, 42, 43]. One day after I/R in males, the number of afferents responsive to *both* noxious (“high metabolite:” pH 6.6, high lactic acid and ATP concentrations, similar to what is produced by the muscle in ischemia [40, 44, 45]) and non-noxious (“low metabolite:” pH 7.0, low lactic acid and ATP concentrations, similar to muscle output during moderate exercise [46–49]) metabolite mixtures [40] is significantly increased. The population that is responsive only to the low metabolite solution is decreased, when compared with age-matched uninjured males [19, 35]. I/R also decreases mechanical thresholds in group III and IV muscle afferents, corresponding with the observed behavioral phenotype [19, 35, 43]. In this study, we sought to determine how I/R injury altered the response properties of these afferents in young, pre-cycling female mice (21–35 days). Additionally, we examined the molecular and behavioral correlates of this injury condition in females compared to males.

## Methods

### Animals

Swiss Webster mice between 21 and 35 days of age were used in all experimental analyses. Mice were obtained from our in-house colony or direct from Charles River (Wilmington, MA). No differences were detected between these two sources. All mice were provided ad libitum access to food and water and housed in a climate-controlled barrier facility with 12-h light/dark housing. Mice that were received from the supplier were allowed 5–7 days to habituate to our facility prior to any procedures. All experimental procedures were approved by the Cincinnati Children’s Hospital Research Foundation Institutional Animal Care and Use Committee and adhered to NIH Standards of Animal Care and Use under Association for Assessment and Accreditation of Laboratory Animal Care International-approved practices. Animals were anesthetized with 3% isofluorane throughout sterile surgeries, and deeply anesthetized with 100 mg/mL ketamine and 20 mg/mL xylazine for all terminal procedures.

### Ischemia and reperfusion injury (I/R) and sham surgeries

As previously described [19, 35], surgical ischemia and reperfusion injury (I/R) of the right forepaw muscles were performed. Briefly, in anesthetized mice, an incision was made in the upper forelimb and the biceps were slightly retracted to expose the brachial artery proximal to the bifurcation into the ulnar and radial arteries. Connective tissue was loosened from around the vessels, and a 7-0 silk suture was tied around the brachial artery. Incisions were closed and animals were returned to their facility in clean cages. Following a 6-h occlusion period, a second surgery was performed to remove the suture from around the brachial artery. To allow adequate reperfusion time, animals were left to recover for 18 h before undergoing any subsequent analyses. As an additional control, sham surgeries were also performed wherein a suture was placed around the brachial artery but not tied during the initial surgery. As naïve and sham males were no different in behavior, physiology, or mRNA/protein expression in our previous reports [19, 35], and also do not differ in the physiology presented here (see Additional file 1), they have been combined as one comparison group for ease of presentation and enhancement of statistical power. However, female shams were behaviorally different from female naïves (Table 3), and as such were presented separately.

### Ex vivo recording

Our novel ex vivo forepaw muscles/median and ulnar nerves/DRGs/spinal cord electrophysiology preparation was performed exactly as previously described [19, 23, 35, 42]. Mice were transcardially perfused with ice cold, oxygenated (95% O_2_/5% CO_2_) artificial cerebrospinal fluid (aCSF).Then the median and ulnar nerves, forepaw, C6-T2 DRGs, and C6-T2 hemisected spinal cord were carefully dissected so that relevant connections remained intact while undergoing continuous perfusion in an ice cold oxygenated aCSF bath. To allow for direct access to receptive fields (RFs) in the muscle tissue, the skin was removed. The entire preparation was then transferred to a two-chambered recording dish. Suction electrodes were placed on the median and ulnar nerves and the aCSF was slowly warmed to 32 °C. Electrically responsive cells were located with an orthograde search stimulus from the suction electrode, and then the RF was found in the muscle using a concentric electrode. RFs were then stimulated with an increasing series of von Frey filaments (0.07 to 10 g), cold (0 °C) and hot (53 °C) physiological saline, and finally, oxygenated “low” (15 mM lactate, 1 mM ATP, pH 7.0) and “high” (50 mM lactate, 5 mM ATP, pH 6.6) metabolite mixtures to assess mechano-, thermo-, and chemo- sensitivity, respectively. ATP was added to the metabolite solutions immediately before application to prevent degradation. After chemical stimulation, mechanical and thermal responsiveness was re-assessed.

In this study, 143 cells were characterized using ex vivo recording: 50 cells from 12 individual naïve females, 47 cells from 13 I/R females, and 46 cells from 12 naïve/sham males. Group III and IV afferents were not significantly different in our samples and were combined for analysis. Data was captured and stored for offline analysis using Spike2.5 (CED). With analysis of every cell, response phenotypes and mechanical thresholds (if present) were confirmed and peak instantaneous frequencies (IF), used to approximate the maximum rate of action potential generation [50], were obtained for each observed response type. Unlike previous observations in males [19, 35], certain parameters in female muscle afferents were found to differ before and after the application of the metabolite solutions used to assess chemosensitivity. Hence, we performed additional analysis to determine whether sex- or injury-dependent changes in afferent sensitivity were ongoing (cumulative peak IF or minimum threshold across recording for each individual cell) or specific to pre- or post-metabolite stimulation. Similar to previous reports, no differences in response properties were observed between cells obtained at the beginning of the recording session compared to the end [19, 23, 35, 42].

### RNA isolation, reverse transcription, and real-time PCR

Quantitative real-time polymerase chain reaction (PCR) was performed as described previously [19, 51]. Following transcardial perfusion with a 1:1 solution of 0.9% NaCl: RNA-later (Ambion), the ipsilateral forepaw muscles and C7/C8/T1 DRGs were excised from age- and sex-matched naïve or 1-day I/R mice. Qiagen RNeasy kits (Qiagen, Valencia, CA) were used to isolate RNA from both regions, either using the standard protocol (DRG) or using the protocol for fibrous tissues (muscle). RNA concentrations were measured on a Nanodrop spectrometer (Thermo) and 500 μg of total RNA from each sample was treated with DNase I, then Superscript II reverse transcriptase (both Invitrogen, Carlsbad, CA). Each real-time PCR reaction was executed in duplicate using 20 μg cDNA with SYBR Green reagents and analyzed on a Step-One real-time PCR machine (Applied Biosystems, Foster City, CA).

Forward and reverse primer sequences for glyceraldehyde 3-phosphate dehydrogenase (GAPDH), GFRα3, ASIC1, ASIC3, and TRPV1 were obtained from Elitt and colleagues [52]. We have previously reported primer sequences used for NGF, NT-3, artemin, P2X3, and P2Y1 [53], for IL-1r1, IL-1β, P2X5, and GDNF [19], and for IL-6 [54]. For the remaining genes investigated in this study, the forward and reverse primer sequences are as follows: interleukin-6 receptor (IL6R): forward, 5′-CCA CCG TTA CCC TGA TTT G-3′; reverse, 5′-GTG TGT TTC CTG TGG TAG TC-3′; monocyte chemoattractant protein 1 (MCP-1): forward, 5′-CAC CTG CTG CTA CTC ATT C-3′; reverse, 5′-CTA CAG CTT CTT TGG GAC AC-3′; P2X4: forward, 5′-GGA GGC ATC ATG GGT ATC CA-3′; reverse, 5′-GTG GGA GGC AGC TCT GTC A-3′; tumor necrosis factor α (TNFα): forward, 5′-CCT ATG TCT CAG CCT CTT CT-3′; reverse, 5′-GGG AAC TTC TCA TCC CTT TG-3′; TNFα receptor (TNFαR): forward, 5′-TCG GAA AGA AAT GTC CCA GGT GGA-3′; reverse, 5′-TGG AAC TGG TTC TCC TTA CAG CCA-3′; TRPM8: forward, 5′-TCT CAC CAA TGA AGT CCT CAC AGA-3′; reverse, 5′-TTC CAC ATC CAA GTC CTC CCT G-3′.

GAPDH was used as an internal control in both tissues, and the mean target Ct value of each sample was normalized to the mean GAPDH Ct value for that sample (∆Ct). Gene expression changes following injury were detected by calculating ∆∆Ct, whereby the mean target ∆Ct from naïve females was subtracted from the mean target ∆Ct from I/R females. The fold-change and error as difference in means for each target gene were then calculated as 2^∆∆Ct^ (Applied Biosystems). For clarity of presentation, values were converted to percent change where two-fold = 100%.

### Protein isolation and western blotting

Protein isolation and Western blot were performed according to our previously detailed procedures [23, 35, 51, 54]. At 1 day, the right forepaw muscles of three female mice from each condition were excised following transcardial perfusion with 0.9% NaCl. Muscles were then homogenized in lysis buffer containing 1% SDS, 10 mM Tris-HCl (pH 7.4), and protease inhibitors (1 μg/ml pepstatin, 1 μg/ml leupeptin, 1 μg/ml aprotinin, 1 mM sodium orthovanadate and 100 μg/ml phenylmethylsulfonyl fluoride; Sigma-Aldrich). Then, a denaturing buffer containing β-mercaptoethanol and SDS was added to 30 μg of each sample. After boiling for 10 min, the sample solutions were separated on a 12% SDS-PAGE gel. Proteins were transferred overnight at 4 °C to a polyvinylidene difluoride (PVDF) membrane (Millipore), which was then blocked in 1:1 LiCor Odyssey blocking buffer in 0.1 M PB, and processed overnight at 4 °C with primary antibodies for GAPDH (chicken α-GAPDH, 1:2000, ProSci) and IL1β (goat α-IL1β, 1:2000, R&D Systems). Infrared-dye conjugated secondary antibodies [donkey anti-chicken 680 nm (1:20,000) and donkey anti-goat 800 nm (1:15,000)] were then applied for detection on a LiCor Odyssey Imaging System using Image Studio v3.1 (LiCor) with consistent detection settings between runs. ImageJ (NIH) was used for densitometry to quantify protein expression of immunoreactive bands relative to GAPDH, and optical density represented as fold-change (mean ± SEM).

### Behavioral analyses

As previously described [19, 23, 35], behavioral analyses of evoked and spontaneous pain-related behaviors were performed by a blinded observer during morning light hours at baseline (BL; immediately before I/R) and days 1, 3, and 5 after I/R (D1, D3, and D5, respectively). Mice were first habituated to a raised acrylic glass chamber with a steel mesh bottom for 30 min. For assessment of ongoing/spontaneous pain, guarding behaviors [55] were assessed for each forelimb every 5 min for 1 h. Guarding scores of 0–2 were thus assigned 12 times based on the following criteria: 0 = full weight-bearing on paw, 1 = weight not firmly distributed on paw, 2 = paw held completely above (not touching) mesh, and averaged for each mouse for analysis of each behavior time point. Evoked mechanical hypersensitivity was quantified using a von Frey paw withdrawal paradigm. For this, the plantar surface of the forepaws was stimulated with an increasing series of calibrated von Frey filaments (0.07–6 g), and paw withdrawal thresholds for each mouse were averaged from three rounds with 5 min between rounds on each behavioral day. Finally, muscle function was tested with a grip strength meter (BioSeb) in three rounds of three trials each with 5 min in home cages between rounds. Mice were held by the tail over the mesh grid of the meter, and once the forepaws, but neither hindpaw, were both firmly grasping the grid, mice were pulled along the axis of the force sensor until they were unable to retain their grip. The nine grip strength measurements (in grams) were then averaged for each mouse on each behavioral day for analysis.

### Statistical analyses

Comparisons of response properties from ex vivo recordings that adhered to a normal distribution were performed with condition x time two-way analysis of variance (ANOVA) with Holm-Sidak, where condition represents either injury or sex, and time refers to pre- or post-metabolite stimulation as this was found to alter response properties in female preparations. Comparisons of non-normal ex vivo mechanical threshold data were analyzed with Kruskal-Wallis one-way ANOVA on ranks followed by Dunn’s post-test, and analysis of afferent phenotype frequency was performed with *χ*^2^ or Fisher’s exact test. All two-way (injury x sex) comparisons of mRNA expression were performed using two-way ANOVAs with Holm-Sidak post hoc analyses on individual ∆Ct values for each gene. To validate gene expression results, within sex comparisons for each gene were made using the Mann-Whitney Rank-Sum test, and all analyses also underwent the false discovery rate procedure to correct for multiple comparisons. Western blot data was examined with one-way ANOVA and Tukey’s test, and behavioral data was analyzed using two-way repeated measures (RM) ANOVA (condition x time) with Holm-Sidak post hoc. Because pain-related behaviors in females who underwent a sham surgical procedure were often found to not differ from either the naïve or I/R conditions, behavioral data are presented here both with (Table 3) and without (Fig. 5) an age-matched sham comparison group.

## Results

### Basal sex differences in mechanical sensitivity and heat responses of primary group III and IV muscle afferents

**Fig. 1:**
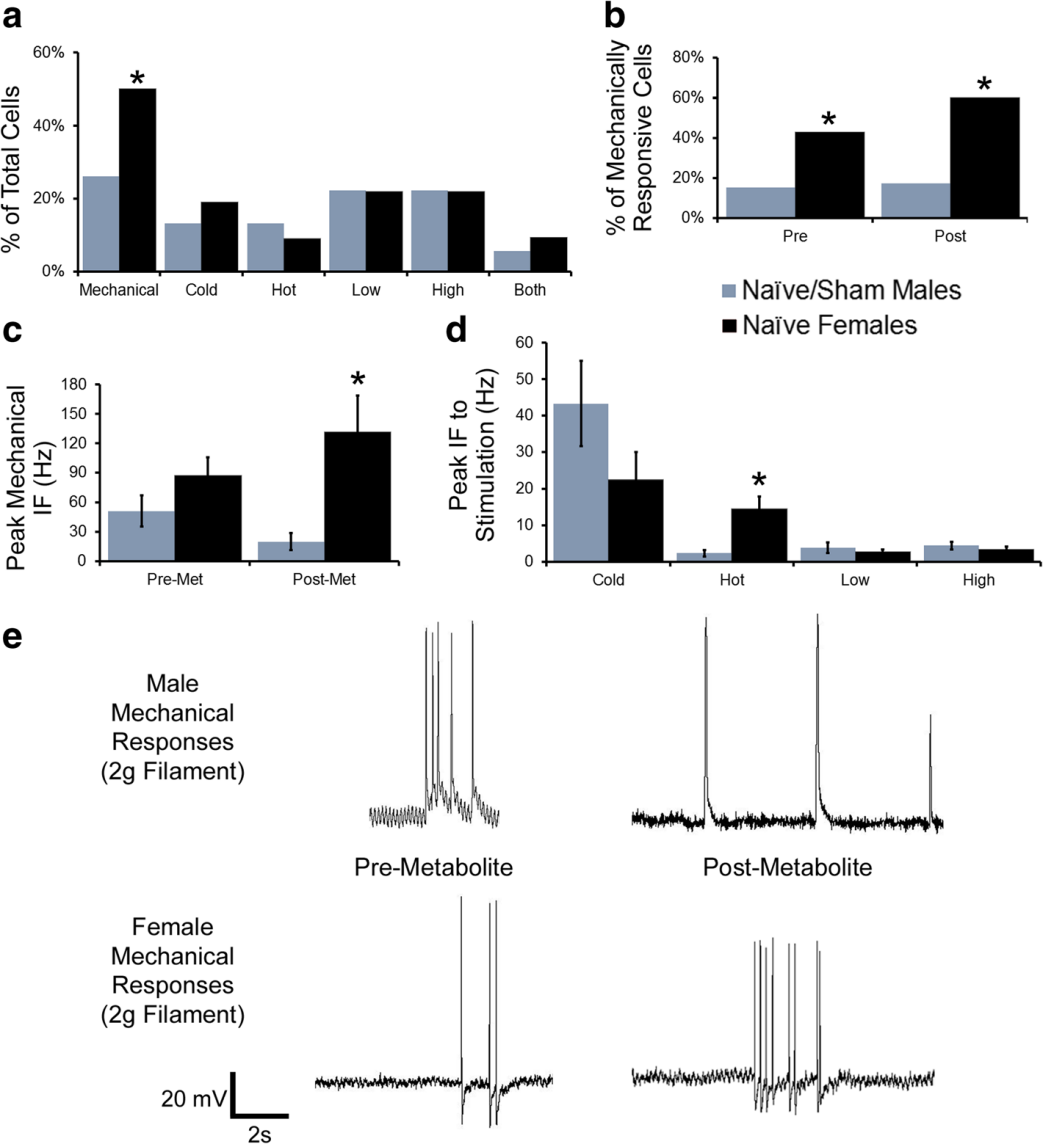
Response properties and phenotypes of individual muscle afferents in age-matched uninjured males and females. **a** Phenotype distribution of male (*n* = 46) and female afferents (*n* = 50) did not differ for most modalities; however, female muscle afferents were more likely to be mechanically sensitive. **b** Sex differences in the numbers of mechanoreceptors was observed both prior to and following the application of metabolite solutions, but firing of these afferents to mechanical stimulation only showed a significant increase in firing after metabolite application **c**. With the exception of post-metabolite mechanical responses and peak heat responses, firing to other stimulus modalities did not differ between males and females (**d**). Representative traces of pre- and post-metabolite mechanical responses to stimulation with a 2 g von Frey filament (**e**). Phenotype data is represented as the percentage of cells responsive to a given stimulus over the total tested in a condition, and the corresponding raw frequency data was analyzed with Fisher’s Exact test. Peak IFs are represented by the condition average ± error of the difference in means.*p < .05 vs. other condition

To provide a stronger foundation for investigating post-injury mechanisms of sensitization in individual group III and IV muscle afferents in females, afferent response properties and phenotypes from naïve females and naïve/sham males were first assessed using our ex vivo muscle/nerve/DRG/spinal cord preparation (Fig. 1). The population distribution of response phenotypes was similar between uninjured males and females, and there were no differences in numbers of cells responsive to cold, hot, or metabolite stimulation. However, female muscle afferents were significantly more likely to respond to mechanical stimulation (males: 12/46, females: 25/50, *p* = 0.021, Fisher’s Exact; Fig. 1a).

As metabolite stimulation may affect responses to other stimuli, we also assessed the frequency of responders to our various stimuli both before and after chemical stimulation. We found that females displayed more mechanically sensitive afferents at both intervals (Fig. 1b; pre-metabolite: males: 7/46, females: 21/49, *p* = 0.004; post-metabolite: males: 6/35, females: 15/25, *p* < 0.001; both Fisher’s Exact). There was no difference in number of metabolite responsive cells (as low/high/both responders/total tested, males: 8/8/2/36, females: 7/7/3/32); however, peak instantaneous frequency to mechanical stimulation was increased in females following metabolite stimulation (Fig. 1c, e; *p* = 0.034, Holm-Sidak post hoc analyses following two way ANOVA with main effect of sex *p* = 0.027), suggesting that the application of metabolite solutions alters firing in mechanically sensitive cells in a sex-dependent manner.

Firing to the low and high metabolite solutions, as well as to cold stimulation was no different between males and females (all *p* > 0.13, one-way ANOVA; Fig. 1d), and no sex differences were detected in mechanical thresholds of individual afferents (pre-met: males: 4.72 ± 2.17 g, females: 3.10 ± 0.82 g; post-met: males: 5.40 ± 2.66 g, females: 3.63 ± 0.76 g, all *p* > 0.05, Kruskal-Wallis ANOVA on Ranks). Interestingly, despite a similarly small proportion of heat-responsive afferents in both sexes (males: 6/45, females: 4/44, *p* = 0.742, Fisher’s Exact; Fig. 1a), the heat-sensitive cells of females were significantly more responsive to heat stimulation (Fig. 1d; males: 2.38 ± 0.91 Hz, females: 14.47 ± 3.47 Hz, *p* = 0.004, one-way ANOVA with Holm-Sidak).

**Fig. 2:**
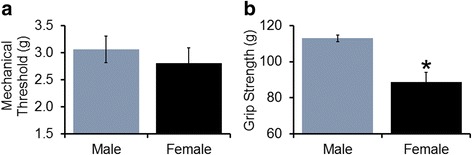
Comparison of baseline behaviors in male and female mice. **a** Male and female mice display similar mechanical withdrawal thresholds upon stimulation of the forepaws. **b** Female mice have significantly reduced grip strength compared to age-matched male mice. *p < 0.003 vs males, one-way ANOVA with Holm-Sidak

We then compared baseline behaviors in male (*n* = 8) and female (*n* = 15) mice in order to determine if alterations in afferents corresponded to any distinctions in sensory-related behaviors. As expected, neither male (0.1 ± 0.04) nor female (0.0 ± 0.02) mice displayed any paw guarding behaviors if uninjured (*p* > 0.05). Additionally, both male (3.1 ± 0.25 g) and female (2.8 ± 0.29 g) mice show similar mechanical withdrawal thresholds (Fig. 2a; *p* > 0.05). However, female (88.8 ± 5.2 g) mice display significantly reduced grip strength compared to age-matched male (113.0 ± 1.9 g) mice (Fig. 2b; *p* < 0.003, one-way ANOVA with Holm-Sidak).

### Transient ischemia and reperfusion injury (I/R) alters group III and IV muscle afferent responsiveness in females

**Fig. 3:**
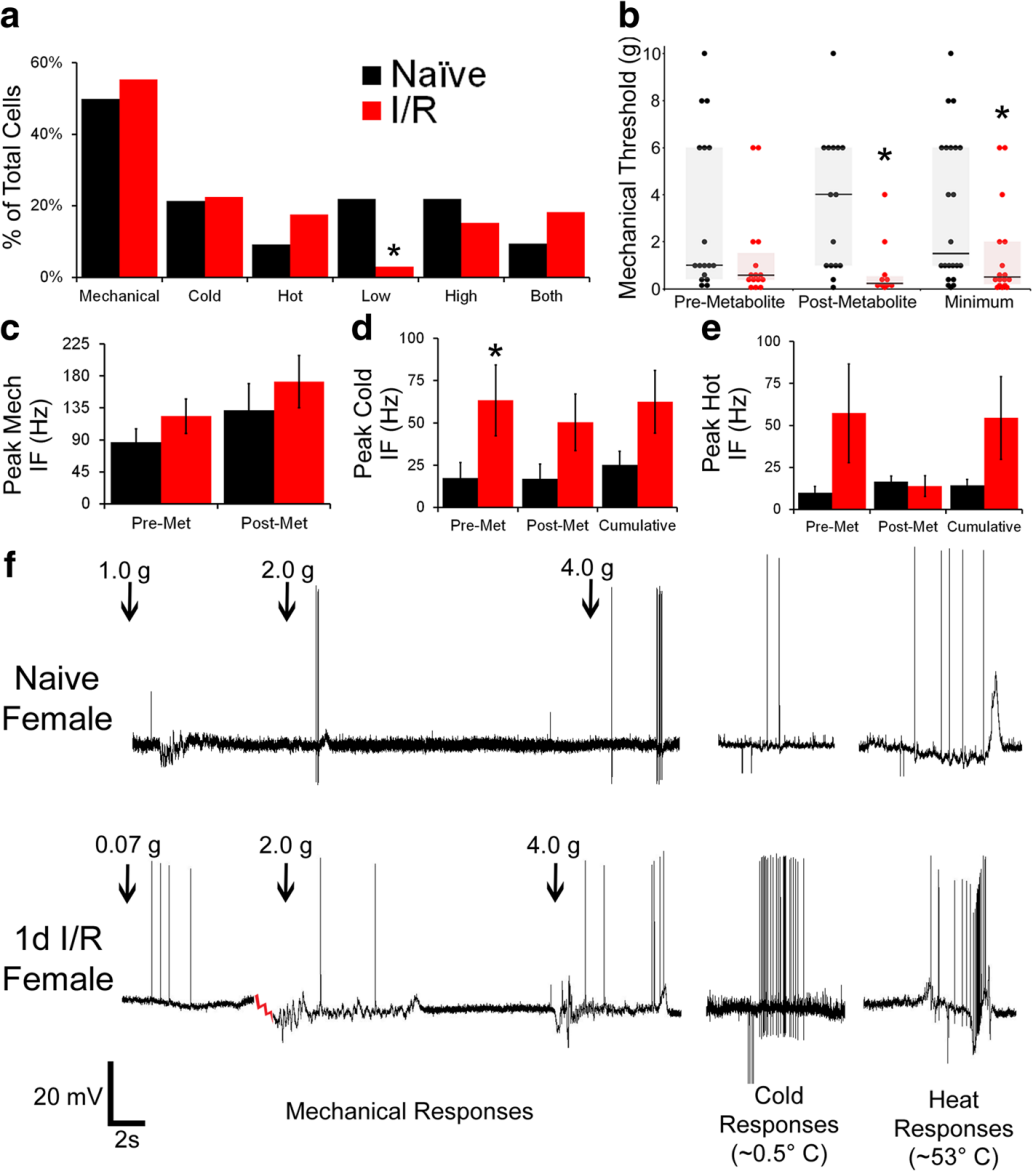
Alterations in response properties and phenotypes of female muscle afferents following I/R. **a** Although minor augmentations in cells responsive to heat or both metabolite solutions were observed, the only significant I/R-evoked difference in afferent phenotype distribution in females was a decreased population of cells that were only responsive to the low metabolite solution. **b** I/R also was found to decrease the mechanical threshold of individual mechanoreceptors, without altering mean peak instantaneous frequencies (IF) to mechanical stimulation (**c**). Increased firing to cold stimulation was observed following I/R in females (**d**), and a similar, but nonsignificant, trend toward I/R-evoked increased heat IF was also seen (**e**). Representative traces from naïve and 1d I/R ex vivo preparations showing responses to mechanical and thermal stimulation (**f**). Phenotype data is represented as the percentage of cells responsive to a given stimulus over the total tested in a condition. Mechanical thresholds of individual neurons are plotted with median and interquartile range demarcated. Peak IF is represented by the condition average ± error of the difference in means.*p < .05 between conditions

Our ex vivo muscle preparation was also used to characterize afferent response properties and phenotypes 1d following transient ischemia and reperfusion injury (I/R) in female mice, which we have thoroughly examined in males [19, 35]. The prevalence of mechanically and thermally sensitive fibers was not affected by I/R; but the number of cells responsive to low metabolite solutions was significantly decreased following this injury (Fig. 3a; naïve: 7/32, I/R: 1/33; *p* = 0.021, Fisher’s Exact). Additionally, I/R females showed decreased thresholds to mechanical stimulation, particularly following metabolite exposure [Fig. 3b, f; Pre-met Threshold (median {Q_1_:Q_3_}): naïve: 1.0 {0.6:6} g, I/R: 0.6 {0.4:1.5} g, *p* = 0.064; post-met threshold: naïve: 4.0 {1:6} g, I/R: 0.28 {0.16:0.55} g, *p* = 0.004; minimum threshold: naïve: 1.5 {1:6} g, I/R: 0.5 {0.22:2} g, *p* = 0.045; all Kruskal-Wallis ANOVA on Ranks], but firing to mechanical stimulation was no different compared to naïve females (Fig. 3c, f).

I/R also altered the responses of individual muscle afferents to thermal stimulation. When compared with age-matched naïve cold responders, firing to cold stimulation was increased by I/R, and was particularly enhanced prior to metabolite exposure (Fig. 3d, f; Pre-met Peak IF: naïve: 17.5 ± 9.1 Hz, I/R: 63.3 ± 20.8 Hz; post-met peak IF: naïve: 16.9 ± 8.9 Hz, I/R: 50.4 ± 16.6 Hz; cumulative peak IF: naïve: 25.2 ± 8.0 Hz, I/R: 62.4 ± 18.5 Hz; two-way ANOVA condition x time with main effect of condition *p* = 0.021 and Holm-Sidak post hoc condition within pre-met *p* = 0.048). Heat responses showed a similar pattern; however, the small number of cells responsive to heat (naïve: 4/44, I/R: 7/40) limited statistical power, thus I/R effects on firing of heat-sensitive muscle afferents in females are inconclusive (Fig. 3e, f; Pre-met Peak IF: naïve: 9.9 ± 5.2 Hz, I/R: 57.2 ± 29.3 Hz; Post-met Peak IF: naïve: 16.6 ± 3.4 Hz, I/R: 13.8 ± 6.2 Hz; Cumulative Peak IF: naïve: 14.5 ± 3.5 Hz, I/R: 54.4 ± 24.6 Hz). Unlike previous phenotype differences in I/R males [19], the proportions of cells responsive to one, two, or three or more modalities did not differ between naïve and I/R females (data not shown).

### I/R induces sex-dependent Upregulation of sensory receptors despite similar increase in muscle IL1β

We have previously documented [19, 23, 35] that ischemic injury evokes dynamic upregulation of sensory-related receptors within the affected DRGs in males, which correlated with increased muscle afferent sensitivity and in vivo pain-related behaviors. I/R injury, specifically, resulted in enhanced IL1β within the muscle that acted at its upregulated receptor, IL1r1, within the DRGs to stimulate de novo ASIC3 expression [19, 35]. Here, we sought to investigate whether altered IL1β/IL1r1 signaling or similar mechanisms following I/R correspond with the observed injury-related afferent physiology changes in females. To that end, mRNA expression levels of genes related to male I/R-evoked hypersensitivity were assessed in naïve and I/R mice of both sexes using qPCR. Validating our past results [19, 35], males showed I/R evoked increases in acid-sensing ion channels (ASICs) 1 and 3, as well as the interleukin-1 receptor (IL1r1), and the purinergic receptors P2X3 and P2X5 within the affected DRGs; however, of these receptors, only IL1r1 and P2X3 were upregulated in females (Table 1). Additionally, whereas males show no I/R-related increase in the transient receptor potential family members that participate in cold and heat sensation, TRPM8 and TRPV1, respectively, this same injury evoked a substantial increase in both TRPM8 and TRPV1 in females.

**Table 1:** Percent changes in mRNA from C7/C8/T1 DRGs in male and female mice

Gene	Naïve female expression	I/R-induced upregulation		Two-way comparisons		
	(Relative to naïve males)	Male	Female	Injury	Sex	Interaction
ASIC1	15.3 ± 17%	77.1 ± 13% *	34.8 ± 17%	< 0.001	0.955	0.193
ASIC3	− 9.7 ± 12%	85.0 ± 9% *	10.6 ± 11%	< 0.001	< 0.001	0.002
IL1r1	− 10.1 ± 17%	74.0 ± 10% *	95.0 ± 23% *	< 0.001	0.698	0.659
P2X3	− 59.7 ± 29% *	75.8 ± 23% *	181.9 ± 21%*	< 0.001	< 0.001	0.112
P2X5	− 33.7 ± 18%	50.2 ± 8% *	63.2 ± 22% *	< 0.001	0.004	0.740
TRPM8	− 46.4 ± 20% *	17.8 ± 19%	93.4 ± 24% *	0.005	0.01	0.085
TRPV1	− 40.1 ± 14% *	42.3 ± 23%	133.7 ± 17% *	< 0.001	0.078	0.098

Values indicate percentage change in mRNA expression of naïve females relative to naïve males and 1d I/R animals of both sexes relative to sex- and age-matched naïve controls ± error of the difference in means (*n* = 4–11 per condition). Final * *P* < .05 obtained via false discovery rate correction following two-way ANOVA on raw sample ∆Cts

To further characterize DRG gene expression in females, we tested an additional panel of sensory-related receptors. Like previous observations in males [19], we found that I/R did not change the expression of the artemin-responsive growth factor receptor, GDNF family receptor α3 (GFRα3) or the ADP-receptor P2Y1, but led to increased P2X4 mRNA expression (Table 2). Furthermore, naïve and I/R females did not differ in expression of receptors for the cytokines tumor necrosis factor α (TNFαR) and interleukin-6 (IL6r).

**Table 2:** I/R-induced changes in gene expression in female forepaw muscles and C7/C8/T1 DRGs relative to naïves

Gene	DRG expression	Gene	Muscle expression
GFRα3	4.5 ± 24%	Artemin	− 38.1 ± 43%
IL6R	− 20.8 ± 46%	GDNF	42.5 ± 26%
P2X4	108.3 ± 15% *	IL-1β	− 7.6 ± 62%
P2Y1	− 6.3 ± 22%	IL-6	− 44.3 ± 53%
TNFαR	− 20.7 ± 45%	MCP-1	53.4 ± 75%
		NGF	− 14.6 ± 15%
		NT-3	− 10.5 ± 25%
		TNFα	− 2.5 ± 29%

Values indicate percentage change relative to sex- and age-matched naïve controls ± error of the difference in means (*n* = 6–11 per condition). *P < .05 vs. naïve obtained via Mann-Whitney Rank-Sum tests for each gene on raw sample ∆Cts, followed by the false discovery rate multiple comparisons correction within each tissue type

**Fig. 4:**
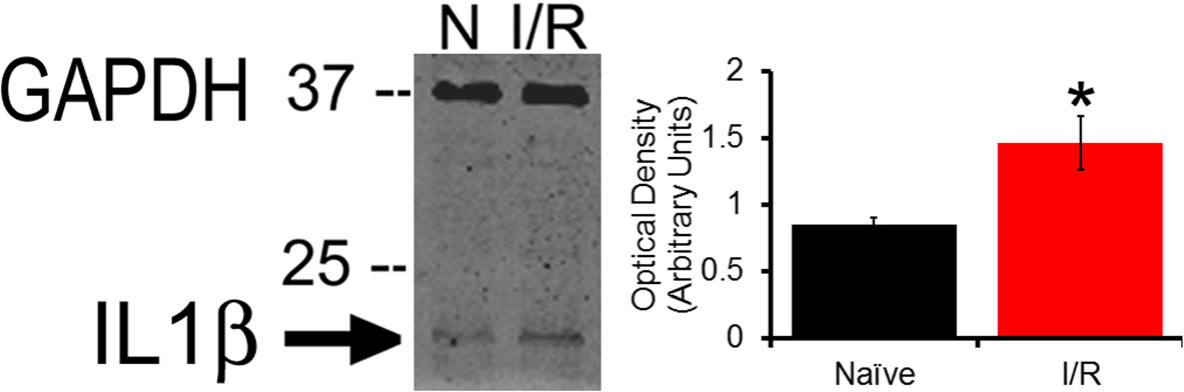
Increased muscle IL1β following I/R in females. Muscles were excised and protein isolated from age-matched naïve and 1 day I/R females. Shown are a representative blot and quantification data of IL1β normalized to GAPDH. Optical density is represented by the condition average ± error of the difference in means where *n* = 3.*p < .05 between conditions as assessed by one-way ANOVA with Tukey’s test

Additionally, mRNA expression of neurotrophic factors, growth factors, and cytokines within the muscle tissue was compared between naïve and I/R females (Table 2); however, none of the tested factors were found to be significantly enhanced at the mRNA level. Because both sexes showed upregulation of IL1r1 mRNA following I/R, we elected to further examine muscle IL1β via Western blot (Fig. 4), finding that IL1β protein is indeed elevated in females 1d following I/R as compared with naïves (optical density in arbitrary units; naïve: 0.854 ± 0.05, I/R: 1.464 ± 0.20; *p* = 0.045, one-way ANOVA with Tukey’s post hoc).

### I/R increases pain-related behaviors

**Fig. 5:**
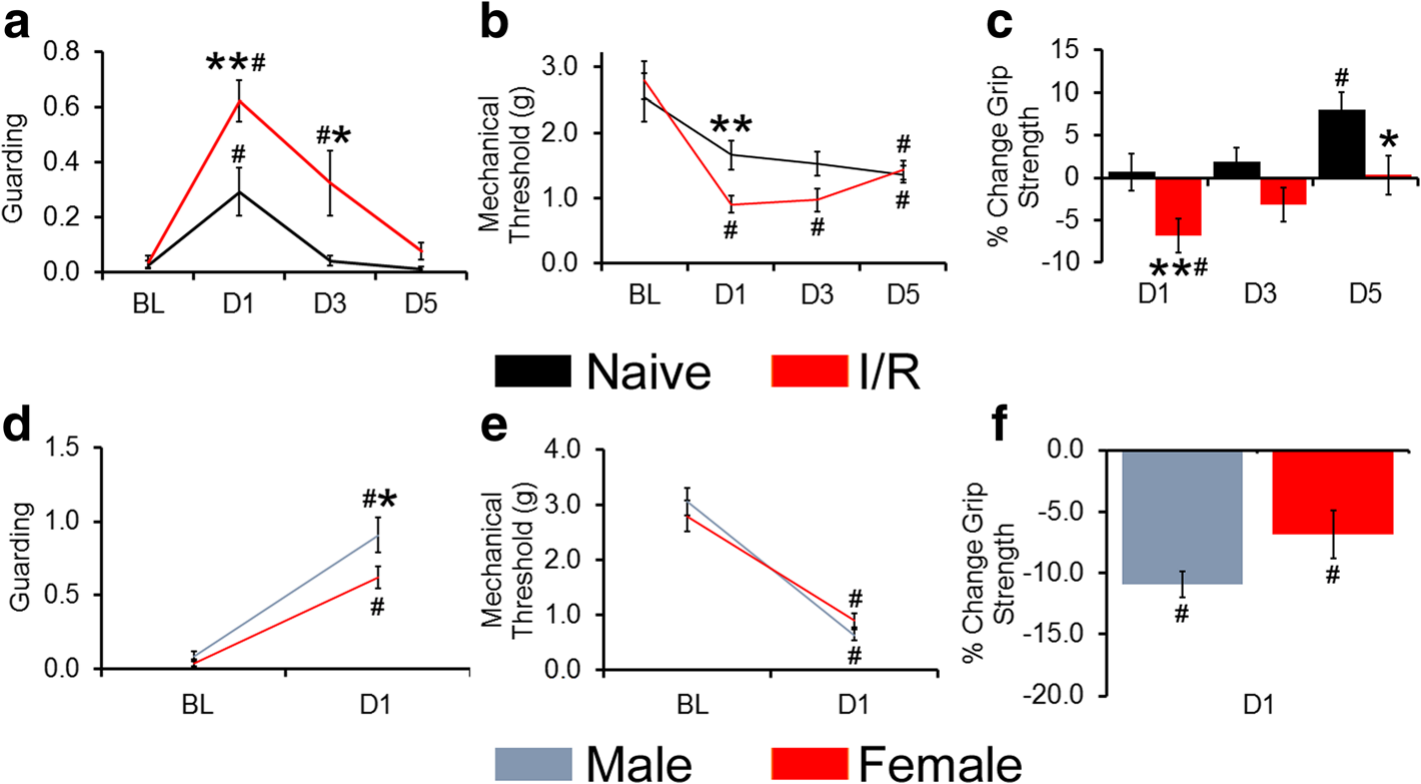
Assessments of pain-like behaviors in naïve females and those with I/R injury. **a** Average guarding scores from the ipsilateral forelimb were increased at D1 in both naïve and I/R animals, but the greatly enhanced guarding behaviors in I/R persisted until the D5 measurements. **b** I/R evoked a substantial decrease in mechanical withdrawal threshold that was restored to naïve levels on D5. **c** Grip strength, an assessment of evoked muscle function, was also significantly decreased with I/R injury. **d** Both male and female mice display increased guarding behaviors at D1 after I/R, but males also show higher guarding scores than females at this time point. **e** Equally reduced mechanical thresholds are observed in male and female mice with I/R at D1. **f** I/R also causes a similar reduction in grip strength in male and female mice at D1. All data are represented as condition average ± error of the difference in means, where grip strength measurements are presented as individual percentage change from BL, and analysis was performed using two-way RM ANOVA with Holm-Sidak. *p < .05 between conditions, ** *p* < .01 between conditions, #*p* < 0.01 vs within condition BL

In male mice, I/R was previously observed to alter three pain-related behaviors that together allow us to observe discrete facets of muscle pain in our animal injury model when also assessing afferent sensitization [19, 35]. Here, these behaviors were also assessed in females at baseline (BL) and 1, 3, and 5 days following I/R injury (D1, D3, D5, respectively). To assay spontaneous or ongoing pain, we observed forelimb guarding over the course of 1 h (Fig. 5a). Interestingly, guarding scores of both naïve and I/R mice were significantly increased at D1 compared with their respective BLs (naïve: BL: 0.03 ± 0.02, D1: 0.29 ± 0.09, *p* = 0.009; I/R: BL: 0.04 ± 0.02; D1: 0.62 ± 0.07, *p* < 0.001). Regardless, I/R female guarding scores were significantly higher than age-matched naïves at D1 and D3, and not at BL or D5 (D3 I/R: 0.32 ± 0.12; two-way RM ANOVA condition x time with Holm-Sidak, interaction effect *p* = 0.008). Naïve guarding scores on D3 and D5 did not differ from BL values (Naïve D3: 0.04 ± 0.02, D5: 0.01 ± 0.01), and the I/R-evoked increase in guarding was restored to BL/naïve levels at D5 (I/R: 0.03 ± 0.01).

Mechanical withdrawal thresholds were then tested by stimulating the forepaws with an increasing series of von Frey filaments (0.07 g–6 g; Fig. 5b). Naïve and I/R females did not differ in BL mechanical threshold (naïve: 2.53 ± 0.37 g; I/R: 2.80 ± 0.29 g, *p* = 0.515), but only I/R mice experienced a threshold decrease at D1 (naïve: 1.66 ± .22 g, *p* = 0.094 vs. BL; I/R: 0.90 ± 0.13 g, *p* < 0.001 vs. BL), the sole time point wherein I/R thresholds were significantly lower than naïves (D1 *p* < 0.001, D3/D5 naïve vs. I/R *p* ≥ 0.18). For I/R mice, thresholds were decreased from BL on all testing days (D3: 0.97 ± 0.18 g, *p* < 0.001; D5: 1.44 ± 0.15 g, *p* = 0.007), whereas naïve females experienced a significant decrease from their respective BL values at D5 (D3: 1.52 ± 0.18, *p* = 0.25; D5: 1.37 ± 0.13, *p* = 0.037).

Finally, grip strength was measured as a means of testing forepaw muscle function (Fig. 5c). D1 grip strength was found to decrease from BL in I/R mice (BL: 88.4 ± 5.2 g, D1: 78.5 ± 5.8 g, *p* = 0.006); however, changes in naïve females were not detected until D5, when grip strength was significantly enhanced over naïve BL and D1 assessments (BL: 78.8 ± 5.1 g, D1: 79.8 ± 5.2 g, D5: 96.7 ± 4.9 g, *p* = 0.006 vs. BL, *p* = 0.021 vs. D1). I/R evoked grip strength decrement differed from naïves at D1 (naïve: 0.68 ± 2.2%, I/R: − 6.82 ± 2.0%, *p* = 0.008) and D5 (naïve: 8.03 ± 2.0%, I/R: 0.30 ± 2.3%, *p* = 0.026), but not at D3 (naïve: 1.90 ± 1.6%, I/R: − 3.18 ± 2.1%, *p* = 0.242).

These three behaviors were also assessed at the same time points in age-matched females that had undergone a sham surgical procedure, and unlike in male sham animals [19, 35], there were behavioral differences from naïves; hence this data has been separately analyzed in Table 3 to prevent statistical dilution during analysis of I/R-induced ischemic myalgia-like behaviors in females. Although sham females showed similar outcomes on BL and D1 guarding to naïve females, similar to I/R females, sham guarding scores remained elevated relative to BL measurements on D3. Furthermore, they did not differ from I/R females in measurements of grip strength. In assessment of mechanical withdrawal thresholds, sham females showed significantly lower BL thresholds than both the naïve and I/R groups, but they were no different from either group at any other time point.

**Table 3:** Raw behavioral assessment data from females with sham or I/R surgery and uninjured females

	Behavioral assessments							
Condition	Guarding score (ipsilateral)				Guarding score (contralateral)			
	BL	D1	D3	D5	BL	D1	D3	D5
*Naïve*	0.03 ± 0.02 (12)	0.29 ± 0.09 (12)^#^	0.04 ± 0.02 (6)**	0.01 ± 0.01 (8)	0.0 ± 0.0 (12)	0.08 ± 0.03 (12)	0.04 ± 0.04 (6)**	0.05 ± 0.05 (8)
*Sham*	0.0 ± 0.0 (12)	0.40 ± 0.09 (12)^#^	0.23 ± 0.06 (7)	0.05 ± 0.02 (8)	0.01 ± 0.01 (12)	0.11 ± 0.05 (12)	0.21 ± 0.07 (7)	0.02 ± 0.01 (8)
*I/R*	0.04 ± 0.02 (15)	0.62 ± 0.07 (15)^#^*	0.32 ± 0.12 (9)^#^	0.08 ± 0.03 (11)	0.03 ± 0.01 (15)	0.22 ± 0.04 (15)*	0.23 ± 0.08 (9)^#^	0.05 ± 0.02 (11)
Condition	von Frey PWT (g)				Grip Strength (g)			
	BL	D1	D3	D5	BL	D1	D3	D5
*Naïve*	2.53 ± 0.37 (12)	1.66 ± 0.22 (12)^#^**	1.52 ± 0.18 (6)^#^	1.37 ± 0.13 (8)^#^	78.8 ± 5.1 (12)	79.8 ± 5.2 (12)	87.64 ± 6.4 (6)	96.7 ± 4.9 (8)^#^
*Sham*	1.72 ± 0.24 (12)*	0.97 ± 0.18 (12)	1.17 ± 0.18 (7)	1.46 ± 0.14 (8)	86.6 ± 6.5 (12)	80.8 ± 7.2 (12)	87.5 ± 5.4 (7)	95.7 ± 4.1 (8)
*I/R*	2.80 ± 0.29 (15)	0.90 ± 0.13 (15)^#^	0.97 ± 0.18 (9)^#^	1.44 ± 0.15 (11)^#^	88.8 ± 5.2 (15)	78.5 ± 5.8 (15)^#^	92.2 ± 6.1 (9)	93.9 ± 4.3 (11)

Values are presented as mean ± SEM (N), and analysis was performed using two-way RM ANOVA on condition x time. For ipsilateral forepaw guarding, main effects of time (*p* < 0.001) and injury (*p* = 0.008), and a group x time interaction (*p* = 0.038) were found. For von Frey, a main effect of time (*p* < 0.001) and an interaction was found (*p* = 0.015). For grip strength, a main effect of time was found (*p* < 0.001). Holm-Sidak post hoc analyses indicated *p < 0.05 vs other conditions, **p < 0.05 vs I/R, ^#^*p* < 0.01 vs within condition BL. PWT: paw withdrawal threshold

To directly compare males and females with I/R and confirm previous reports [19, 23, 35], we also assessed BL and D1 pain-related behaviors in both sexes. Both male (0.9 ± 0.12) and female (0.6 ± 0.07) mice with I/R showed increased ipsilateral paw guarding (*p* < 0.01) compared to individual BL measurements; however, males displayed slightly but statistically increased D1 scores compared to females (Fig. 5d; *p* < 0.05). I/R injury was found to reduce mechanical withdrawal thresholds in males (0.6 ± 0.1 g) and females ((0.9 ± 0.1 g) at D1 (*p* < 0.01) but this was not found to be different between sexes (Fig. 5e; *p* > 0.05). Finally, although we previously found that males and females display significant differences in BL grip strength (see Fig. 2b), both male (− 10.9 ± 1.05%) and female (− 6.8 ± 1.97%) mice display a similar grip strength decrement at D1 following I/R relative to BL measurements (Fig. 5f; *p* < 0.05).

## Discussion

Females and males show differing pain tolerance and prevalence of pain conditions in clinical reports [29, 56–58]. Sex-dependent pain mechanisms have also been described in rodent models [36, 37, 39, 59]. However, the physiological effects of sex-dependent primary afferent sensitization have yet to be understood, particularly in regard to muscle nociceptors. In this study, we first compared the response properties and phenotypes of individual group III and IV muscle afferents in age-matched uninjured males and females, which yielded basal sex differences (Fig. 1). Notably, the population of mechanically sensitive afferents was significantly greater in females, but unlike previous recordings of nociceptors in vitro [60], no sex difference was detected in the mechanical thresholds of individual muscle afferents. Interestingly, the peak IF to mechanical stimulation was increased in females compared to males, particularly following metabolite stimulation, even though there was no difference in proportion of metabolite-sensitive cells or responsiveness to metabolite solutions. This suggests that metabolite stimulation may potentiate mechanoreceptor responses under basal conditions in females, which has not been previously documented in males [19, 35]. This may be one reason why females display reduced grip strength at baseline compared to males (Fig. 2), since grip-induced muscle contractions would produce the metabolites used in ex vivo recordings (e.g., [40]); however, this will need to be confirmed in future studies.

Basal sex differences in nociceptor response properties may originate from altered sensitivity or expression of pain- and sensory-related receptors. While changes in metabolite responsiveness are often linked to DRG upregulation of ASIC3, P2X3, and TRPV1 [40, 41], basal expression of TRPV1 and P2X3 mRNA was found to be significantly lower in females than in males, while ASIC3 did not differ between the sexes (Table 1). The increased firing to heat in females may suggest sex differences in TRPV1 [61, 62], a known heat transducer. However, TRPV1 was also found in this study to undergo I/R-induced upregulation in females (Table 2) *without* significantly enhancing post-injury heat responses (Fig. 3). Thus, other modifications in TRPV1 (or other channels not tested) such as enhanced sensitivity or activation may be more likely explanations than increased mRNA expression for female-specific potentiation of muscle afferent heat responses. It is important to note however that gene expression was obtained from whole DRGs and not from muscle afferents specifically.

Additionally, synergistic interactions between P2X3 and TRPV1 have been linked to peripheral afferent sensitization in a variety of models [63–65]. Females experience dynamic changes in DRG P2X3 expression throughout life [66, 67] and have unique P2X3 responses to ATP [68–70]; hence, this channel and its interactions may be particularly relevant in understanding sex effects on muscle afferent responses. Individual muscle afferents are unlikely to respond to ATP alone in culture [40], but ATP action at P2X3 has been long known to potentiate chemical responses in nociceptors [41, 71, 72]. Despite the presence of I/R-induced P2X3 upregulation in both sexes (Table 1), ATP-responsive afferents have not been observed in our previous studies of ischemic injury in males [19, 23, 35], suggesting that sex-specific contributions of P2X3 in particular may also be relevant to discrete mechanisms of injury-evoked sensitization that necessitate further investigation.

Like previous results from analysis of I/R in males [19, 35], electrophysiological recordings from females exhibited fewer chemosensitive cells responsive to only an innocuous, but not a noxious, metabolite solution (low responders), and decreased mechanical thresholds in individual mechanoreceptors (Fig. 3) 1 day following I/R when compared with sex- and age-matched naïves. Although I/R males have consistently displayed more afferents responsive to both metabolite solutions than naïve males, no single response modality was found to be significantly increased following female I/R, suggesting that female group III and IV muscle afferent sensitization proceeds through differing, potentially more subtle, mechanisms [19, 35].

Additionally, females with I/R also showed substantial increases in firing to thermal stimulation, which correlates with TRPV1 and TRPM8 upregulation within the affected DRGs (Table 1). Males have not been found to experience enhancements of either of these channels following I/R [19, 35]; however, P2X4, another channel linked to cold sensitivity following injury [73], was upregulated in both sexes with 1d I/R [19]. Furthermore, the robust ASIC3 increase induced by IL1β action at IL1r1 in males [35] is absent in females (Table 1), suggesting that sex-dependent alterations in gene expression may underlie differential injury-induced changes in the response properties and phenotypes of individual muscle afferents.

Despite lacking an enhancement in muscle IL1β mRNA, IL1β protein expression in muscles is significantly increased 1d following I/R in females (Fig. 4). As we found in our previous study of male muscle afferents that IL1β treatment induced TRPV1 upregulation in vitro [35], it is possible that the observed I/R-induced increase in DRG TRPV1 in females may also result from enhanced IL1β. Furthermore, effects of IL1β on female sensory afferents may not be limited to changes in gene expression. A previous study by Obreja and colleagues showed that just 1.5 min exposure to 20 ng/mL IL1β was sufficient to sensitize cultured female DRG neurons to noxious heat stimulation, likely through activation of PKC [74]. Altogether this suggests that IL1β may modulate I/R-evoked muscle afferent sensitization in females in a variety of ways that may differ from the previous IL1r1-mediated mechanism characterized in males. Further supporting this notion, the IL1 receptor antagonist, which potently inhibits IL1β/IL1r1 signaling, was recently found to be ineffective at reducing pain in the treatment of women with chronic fatigue syndrome, which has also been linked to insufficient peripheral perfusion [75]. Regardless, the discrepancy between mRNA and protein expression of IL1β in female muscle following I/R may indicate a slower degradation rate of IL1β protein in females and/or sex differences in the time course of immune infiltration/cytokine upregulation following this type of injury.

Due to the sex- and injury-dependent differences observed within individual muscle afferents, we wanted to confirm that the assays of pain-related behaviors used to describe an ischemic myalgia-like phenotype in male mice with I/R [19, 35] would also be appropriate to detect this phenotype in female mice. While individually, these assessments do not directly test muscle specific functions, a combination of these measures along with ex vivo recording provide information about the animals’ deep tissue “pain.” The overall pattern of I/R-evoked changes in female pain-related behaviors at D1 was similar to that which had been previously observed in males and confirmed in this report (Fig. 5). Males did, however, display slightly increased guarding at D1 compared to females. Nevertheless, these behaviors, including increased paw guarding, decreased mechanical withdrawal thresholds, and decreased grip strength, were all restored to individual BL or age-matched naïve levels within 5 days after I/R in females [19]. All of these tests have been used because they recapitulate commonly experienced clinical symptoms of ischemic myalgia; however, this approach may have limited the ability to fully correlate physiological and behavioral effects, particularly regarding the altered thermal responses observed both with and without I/R in female mice.

Although the experiments performed here were done in a manner similar to our previous work in males, where we had not observed behavioral changes in uninjured animals, both naïve and sham females also experienced enhanced paw guarding. Furthermore, whereas naïve and sham males have never been found to differ in any assay performed in our previous I/R studies [19, 35], nor in the electrophysiological data presented here (see Additional file 1), sham females exhibited behavioral characteristics distinct from both naïve and I/R females (Table 3), suggesting a possible female-specific enhancement of incision or surgical pain following sham surgery that is undetectable in males. Because this typically non-noxious surgery still altered this single pain-related behavior relative to naïves, more investigation will be needed to determine both what may be underlying these changes and if an alternative sham procedure, such as isolating the artery without placing a suture around it for the duration of the injury, may serve as a more inclusive control in studies of both sexes. Interestingly, controlled clinical studies have also documented enhanced post-surgical pain and time to recovery in girls and women as compared to boys and men undergoing similar procedures [33, 76–78]. Altogether, this implies that both naïve and sham controls should be considered for analyses of surgical injury models, particularly in studies of females and/or sex differences.

Recent studies have determined that uninjured rodents, when housed with those who have undergone a painful procedure, may present with similar pain-like behaviors [79–81]. As in our previous work with this injury model, females in this study were socially housed (≤ 4/cage) with injury conditions mixed within cages to allow for adequate blinding; thus, it is possible that the observed increase in guarding behaviors at D1 in naïve and sham females may stem from a similar social transfer. Regardless, this phenomenon was not observed in our previous studies of male mice [19, 35]. Future studies that include females should be performed with this potential cofactor in mind.

## Conclusions

With the comparison of basal contributions of group III and IV primary muscle afferents in age-matched males and females, and characterization of the behavioral, molecular, and physiological correlates of I/R injury in females, this study provides insight into primary sex differences that manifest at the peripheral afferent level. This may not come as a surprise, as sex differences in muscle afferent responses to glutamate have been previously documented in humans and rodents [82]; however, the initial study of the response properties and phenotypes of individual female muscle afferents to natural stimulation at their receptive fields presented here establishes a basic foundation for future research into sex-dependent mechanisms of nociceptive muscle afferent sensitization.

## Additional file

Additional file 1: Comparison of Naïve and Sham male ex vivo response phenotypes. (DOCX 12 kb)
