# Supplementary material for: Sex differences in primary muscle afferent sensitization following ischemia and reperfusion injury
Source: Biol Sex Differ. 2018 Jan 3;9:2. doi: 10.1186/s13293-017-0163-5 (PMC5751812; doi:10.1186/s13293-017-0163-5)
Supplement: Additional file 1: — Comparison of Naïve and Sham male ex vivo response phenotypes. (DOCX 12 kb) [file 13293_2017_163_MOESM1_ESM.docx]

**Additional Material:**

**Additional file 1:** *Comparison of Naïve and Sham male* ex vivo *response phenotypes.*

|  | **Response Counts** | | | | | |
| --- | --- | --- | --- | --- | --- | --- |
| **Modalities** | **Mechanical** | **Cold** | **Hot** | **Low** | **High** | **Both** |
| Naïve | 10/39 | 5/39 | 6/39 | 7/30 | 7/30 | 2/30 |
| Sham | 2/7 | 1/6 | 0/6 | 1/6 | 1/6 | 0/6 |
| *p-Value* | *1.0* | *1.0* | *0.58* | *1.0* | *1.0* | *1.0* |

No difference in proportion of cells responsive to each stimulus type applied during electrophysiological recording of naïve (n=10) and sham (n=2) male *ex vivo* preparations (Fisher’s Exact Test).
